# The efficacy and safety of indocyanine green-hyaluronic acid mixture (LuminoMark™) for localization in patients with non-palpable breast lesions: a multi-center open-label parallel phase-2 clinical trial

**DOI:** 10.1186/s12893-021-01129-y

**Published:** 2021-03-16

**Authors:** Isaac Kim, Hee Jun Choi, Jai Min Ryu, Se Kyung Lee, Jong Han Yu, Jeong Eon Lee, Seok Jin Nam, Hyuk Jai Shin, Seok Won Kim

**Affiliations:** 1 Department of Surgery, Bundang CHA Medical Center, CHA University School of Medicine, 59 Yatap-ro, Bundang-gu, Seongnam-si, Gyeonggi, Republic of Korea; 2 Division of Breast Surgery, Department of Surgery, Samsung Medical Center, Sungkyunkwan University School of Medicine, 81 Irwon-ro, Gangnam-gu, Seoul, 06351 Republic of Korea; 3 Department of Surgery, Myongji Hospital, Hanyang University College of Medicine, Gyeonggi, Republic of Korea

**Keywords:** Breast, Localization, Indocyanine green

## Abstract

**Background:**

Increasing rates of breast cancer screening have been associated with an increasing frequency of non-palpable breast lesions detection. Preoperative breast lesion localization is essential for optimizing excision accuracy. This study aimed to evaluate the efficacy and safety of indocyanine green (ICG) hyaluronic acid injection as a novel mixture for localization.

**Methods:**

We performed a prospective clinical trial with female patients who underwent surgery for non-palpable breast lesions. All patients were sequentially assigned to the control group (localization with activated charcoal), Test Group 1 (ICG-hyaluronic acid mixture 0.1 mL), or Test Group 2 (ICG-hyaluronic acid mixture 0.2 mL) by 1:1:1 ratio.

**Results:**

A total of 44 patients were eligible for this study (Control Group = 14, Test Group 1 = 15, Test Group 2 = 15 patients). Fibroadenoma (n = 17, 38.6%) accounted for the largest proportion of diagnoses, and five patients (11.4%) were diagnosed with malignancies. There were no statistically significant differences in baseline characteristics among the three groups. The marking rate was over 86% in all groups, with no significant intergroup differences. Skin pigmentation was only observed in the control group. The mean accuracy of resection (the greatest diameter of the excised specimen divided by the greatest diameter of the preoperative lesion as observed using ultrasonography, with values closer to 1 reflecting a higher accuracy) was 3.7 in the control group, 2.2 in Test Group 1, and 2.1 in Test Group 2 (p = 0.037 between Controls and Test Group 1, p = 0.744 between Test Group 1 and Test Group 2, and p = 0.026 between Controls and Test Group 2).

**Conclusion:**

ICG-hyaluronic acid injection is a novel method that was shown to accurately localize non-palpable breast lesions and was associated with no skin pigmentation. Further research is required to apply this method to malignant breast lesions.

*Trial registration* “A Multicenter Open-label, Parallel, Phase 2 Clinical Trial to Evaluate the Efficacy and Safety of LuminoMark™ Inj. (Conc. for Fluorescence) Localization in Patients with Non-palpable Breast Lesions” was prospectively registered as a trial (ClinicalTrials. gov Identifier: NCT03743259, date of registration: May 29, 2018, https://clinicaltrials.gov/ct2/show/NCT03743259)

## Backgrounds

Increasing rates of breast cancer screening have been associated with an increasing frequency of non-palpable breast lesion detection [1]. Image-guided localization has been shown to decrease positive margin rates and result in fewer re-excisions [2].

In general, there are several ways for localizing non-palpable breast lesions—for example: skin marking with a pen, wire, or tattoo. Skin marking with a pen under ultrasound (US) guidance is simple to perform, but pen markings can be easily erased and affected by a patient’s movements [3]. Wire localization was the standard technique in the early 2000s, but it had several disadvantages: patient discomfort, wire migration, and interference with surgical approaches [4]. US-guided tattoo localization is an accurate and safe method; however, skin pigmentation is common [5].

Preoperative localization of non-palpable breast lesions is essential for optimizing excision accuracy and margin distance. Indocyanine green (ICG)–hyaluronic acid (LuminoMark™) is a mixture that can be used for accurate preoperative localization with no associated skin pigmentation. This study aimed to evaluate the efficacy and safety of ICG-hyaluronic acid injection as a novel localization technique.

## Methods

In this prospective, multi-center, open-label, parallel clinical trial (ClinicalTrials. gov Identifier: NCT03743259), we screen 50 female patients with non-palpable breast lesions who then underwent surgery at Samsung Medical Center and Myongi Hospital between January 2018 and March 2019. This study was approved by the institutional review boards of Samsung Medical Center (IRB No. 2017–10-094) and Myongi Hospital (IRB No. 2018–08-005). All patients provided written informed consent.

### Study design

**Fig. 1:**
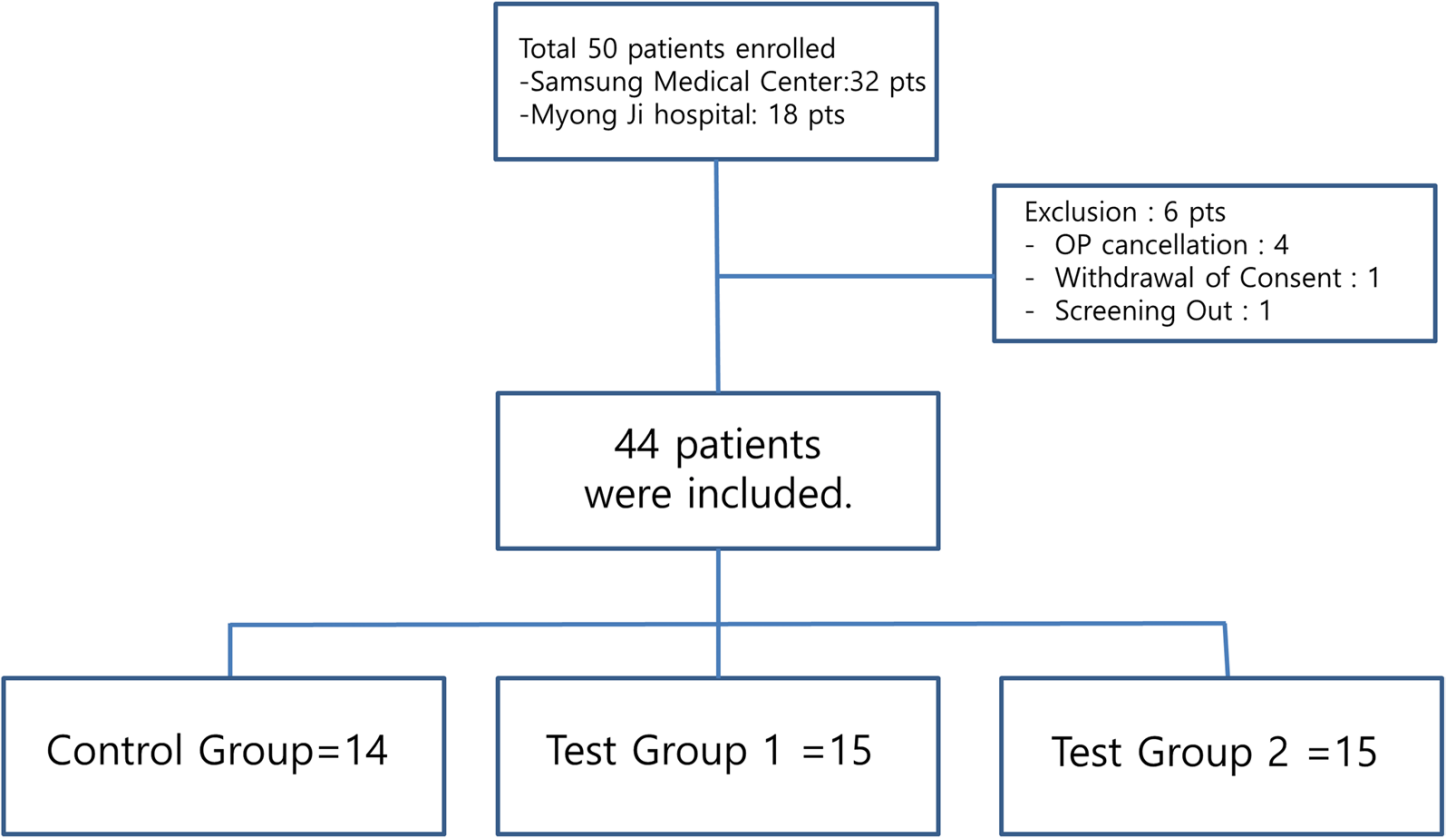
The flow chart of enrollement (Pts:patients, OP:operation)

**Fig. 2:**
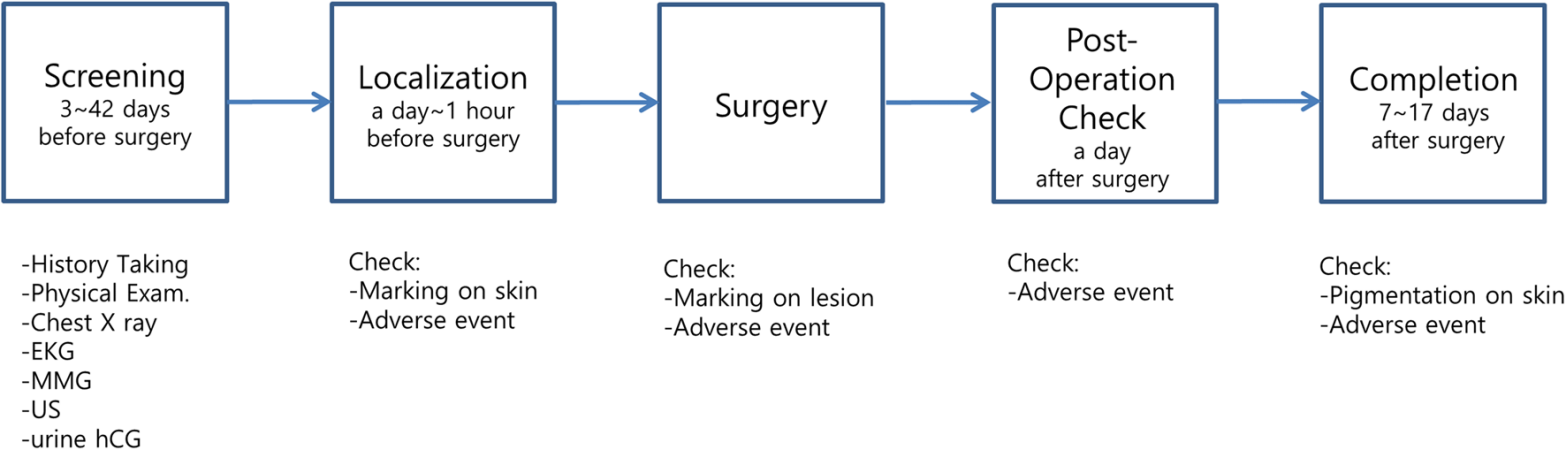
The Trial flow (*Exam* examinatoin, *EKG* electrocardiogram, *MMG* mammography, *US* ultrasound, *hCG* human chorionic gonadotropin)

**Fig. 3:**
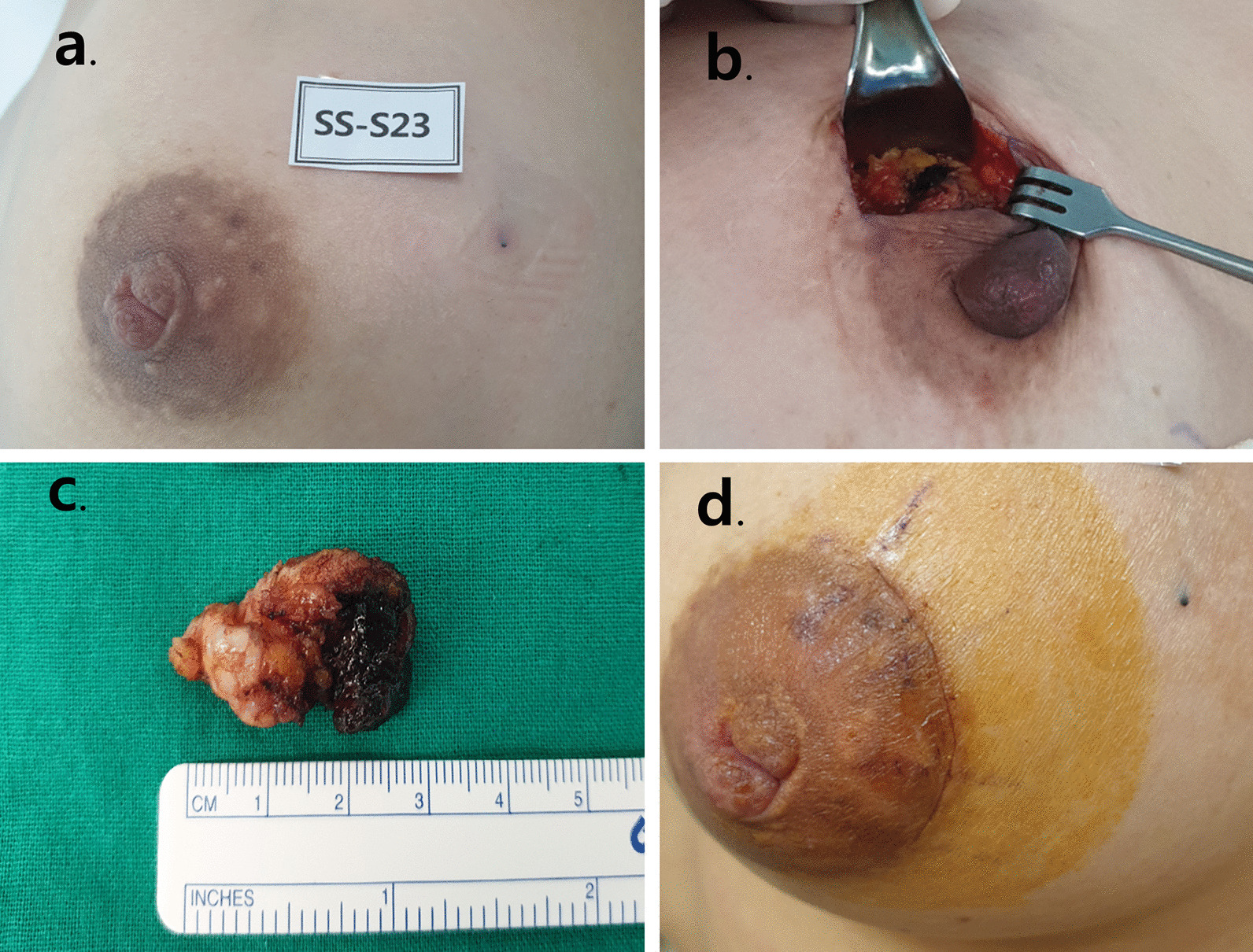
Photos for patient in control group (**a** Before skin incision, **b** after skin incision, **c** after excision, **d** at last follow up day). The injection site was clearly visible by the charcoal

**Fig. 4:**
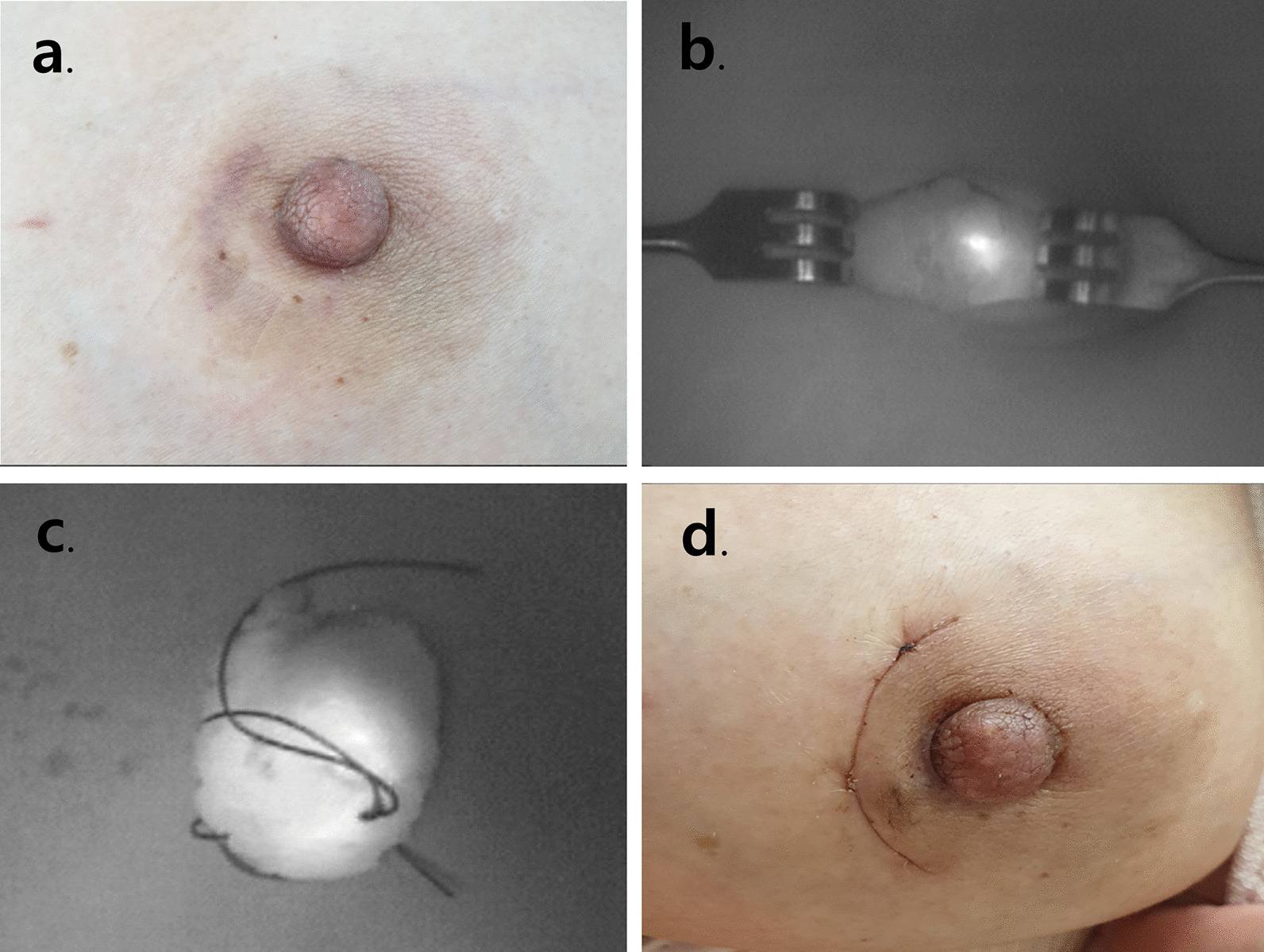
Photos for patient in test group (**a** Before skin incision, **b** after skin incision **c** after excision, **d** at last follow up day). The injection site was invisible

There were 44 eligible patients who were sequentially assigned to the control group, Test Group 1, and Test Group 2 in a 1:1:1 ratio (Fig. 1). Figure 2 shows the trial flow chart. Screening was done from 3 to 42 preoperative days. All patients underwent history taking, physical examination, chest x-ray, echocardiogram (EKG), mammography (MMG), breast US, and urine human chorionic gonadotropin (hCG) testing. A day or an hour before surgery, US-guided localization was performed by any of four dedicated radiologists. For localization, 0.3 to 1.0 mL of activated charcoal (Charcotrace™, 40 mg/mL) was injected through an 18-gauge needle in the control group. Patients in Test Group 1 and Test Group 2 received 0.1 mL and 0.2 mL, respectively, of ICG-hyaluronic acid mixture through 21-gauge needles. The ICG-hyaluronic acid mixture was in the form of a solution mixed using 0.01 mg of ICG and 4 mg/2 mL hyaluronic acid. Localization with activated charcoal was visualized using the naked eye. Localization with ICG-hyaluronic acid was visualized using near-infrared fluorescence. Each excision was conducted by any of five breast surgeons. Intraoperative photographs were captured before the skin incision, after the skin incision, and after excision (Figs. 3 and 4). Each patient underwent a checkup 1 day after surgery, and the last follow-up, including a photograph, occurred between 7 and 17 days after surgery.

### Variables for evaluation

The primary objective of this trial was to evaluate the safety of ICG-hyaluronic acid injection for non-palpable breast lesion localization. We defined all the harmful reactions that occurred after injection or surgery as adverse events (AEs). The primary endpoint was the accuracy of resection, which was defined as the value of the largest diameter (cm) of the excised specimen divided by the greatest diameter (cm) observed on preoperative US assessment.

Secondary endpoints were the success rate of breast lesion marking, marking on the excised specimen, histopathological accuracy, and the presence or absence of pigmentation. The breast lesion marking rate was defined as the percentage of patients identified with localized target lesions for surgery. The excised specimen marking rate was defined as the percentage of patients whose excised specimens were observed as having been stained. The histopathological accuracy was defined as the value of the largest diameter (cm) of the histopathological lesion divided by the greatest diameter (cm) of the excised specimen. Skin pigmentation was defined when pigment remnants were visible on the skin after surgery.

### Inclusion criteria

Patients were eligible if they had non-palpable breast lesions that could be identified by breast US, were over 19 or under 80 years of age, and agreed to participate in the trial.

### Exclusion criteria

Patients were ineligible if they had palpable breast lesions, were under 19 or over 80 years of age, were pregnant, had skin disease (scleroderma or systemic lupus erythematosus, for example), had a history of radiotherapy, neoadjuvant therapy, inflammatory breast cancer, or and drug (charcoal, indocyanine green, hyaluronic acid). Among 50 patients who consented to participating in the trial, six were excluded because of withdrawal of consent, cancellation of surgery, or ineligibility.

### Statistical analysis

The data in this study were analyzed for differences using the chi-square test or Fisher’s exact test. Differences in mean values were assessed using the Mann–Whitney U test. We used SPSS Statistics for Windows, version 19. 0 (IBM Corp., Armonk, NY, USA). *P*-values < 0.05 were considered statistically significant.

## Results

A total of 44 patients were eligible for this study. The control group had 14 patients and each test group had 15 patients. Table 1 shows the baseline characteristics of the included patients. Most were over 40 years old, and there were no significant intergroup differences in age distribution or mean age. According to the histopathologic results, fibroadenoma (n = 17, 38.6%) accounted for the largest proportion of diagnoses, and five patients (11.4%) were diagnosed with malignant breast lesions. Other diagnostic classifications included fibrocystic changes, inflammation, and fat necrosis. The greatest lesion diameters, as determined via preoperative US were mostly under 2 cm, and there were no significant differences among the three groups. The longest excised specimens were mostly between 2 and 3 cm, and there were no significant intergroup differences. The longest excised specimen was 8 cm; this was a malignant lesion excised from a patient in the control group.

**Table 1:** Baseline Characteristics of included Patients (total 44 patients)

	Control Group (*n* = 14)	Test Group 1 (*n* = 15)	Test Group 2 (*n* = 15)	*p-value*
Age(yr)				
<30	0 (0.0%)	0 (0.0%)	3 (20.0%)	*0.143*
30≤ <40	1 (7.1%)	2 (13.3%)	1 (6.7%)	
40≤ <50	5 (35.7%)	9 (60.0%)	7 (46.7%)	
50≤	8 (57.1%)	4 (26.7%)	4 (26.7%)	
mean(range,yr)	52.2(39-68)	47.4(37-57)	442.(26-76)	*0.112*
Pathology				
Fibroadenoma	4 (28.6%)	6 (40.0%)	7 (46.7%)	*0.384*
Phyllodes tumor	0 (0.0%)	1 (6.7%)	1 (6.7%)	
Atypical ductal hyperplasia	4 (28.6%)	0 (0.0%)	0 (0.0%)	
Intraductal papilloma	2 (14.3%)	3 (20.0%)	4 (26.7%)	
Malignancy	1 (7.1%)	2 (13.3%)	2 (13.2%)	
Others	3 (21.4%)	3 (20.0%)	1 (6.7%)	
the longest length on pre OP-US (cm)				
<1	8 (57.1%)	5 (33.3%)	7 (46.7%)	*0.500*
1≤ <2	5 (35.7%)	9 (60.0%)	6 (40.0%)	
2≤	1 (7.1%)	1 (6.7%)	2 (13.3%)	
mean(range,cm)	1.0(0.4-2.7)	1.2(0.6-2.8)	1.3(0.6-2.7)	*0.379*
the longest length of excised specimen (cm)				
<1	0 (0.0%)	1 (6.7%)	1 (6.7%)	*0.253*
1≤ <2	1 (7.1%)	3 (20.0%)	2 (13.3%)	
2≤ <3	6 (42.9%)	7 (46.7%)	7 (46.7%)	
3≤ <4	5 (35.7%)	1 (6.7%)	4 (26.7%)	
4≤	2 (14.3%)	3 (20.0%)	1 (6.7%)	
mean(range,cm)	3.0(1.5-8.0)	2.4(0.8-4.0)	2.5(0.6-6.2)	*0.247*

*yr.* year, *OP* operation, *US* ultra sound

Table 2 shows the accuracy of resection for all patients. The mean accuracy in the control group was 3.7 (range, 1.2–13.3). The mean accuracy values in Test Group 1 and Test Group 2 were 2.2 (range, 1.0–4.2) and 2.1 (range 1.0–4.2), respectively. The control group yielded the highest value (reflecting the lowest accuracy) (p = 0.037 between the control group and Test Group 1, p = 0.026 between the control group and Test Group 2, and p = 0.744 between the test groups).

**Table 2:** The Accuracy of Resection (The primary end point) in all patients

Patients no.	The longest length of excised specimen (cm)	The longest length on pre OP-US (cm)	The accuracy of resection (cm/cm)	Mean (±sd)	*p-value*
Control Group (*n* = 14)					
1	3	1.4	2.1	3.7(±1.5)	*0.037* *(group1-2)*
2	3	0.7	4.3		
3	2.3	1.6	1.4		
4	3	0.8	3.8		
5	2	0.4	5		
6	1.5	0.6	2.5		
7	3.3	2.7	1.2		
8	8	0.6	13.3		
9	2	0.6	3.3		
10	2.5	0.8	3.1		
11	3	1	3		
12	2.8	1.4	2		
13	2	1	2		
14	4	0.9	4.4		
Test Group 1 (*n* = 15)					
1	2.5	1.3	1.9	2.2(±0.5)	*0.744* *(group2-3)*
2	2.5	1.4	1.8		
3	2	1.6	1.3		
4	1.2	0.7	1.7		
5	3.5	2.8	1.3		
6	1.5	1.1	1.4		
7	2	0.9	2.2		
8	2.4	0.8	3		
9	2.5	0.6	4.2		
10	2.5	1.4	1.8		
11	4	1.7	2.4		
12	1.3	1.3	1		
13	4	1.2	3.3		
14	0.8	0.6	1.3		
15	4	1	4		
Test Group 2 (*n* = 15)					
1	2	1.4	1.4	2.1(±0.5)	*0.026* *(group1-3)*
2	6.2	2	3.1		
3	1.2	0.6	2		
4	0.6	0.6	1		
5	2.2	1.7	1.3		
6	1	0.9	1.1		
7	3	0.8	3.8		
8	2.5	1.1	2.3		
9	2.5	0.6	4.2		
10	2.7	1.8	1.5		
11	3.5	2.7	1.3		
12	2.1	1.2	1.8		
13	2.8	0.8	3.5		
14	3.5	1.8	1.9		
15	1.1	0.9	1.2		

*No.* numbers, *OP* operation, *US* ultra sound, *sd* standard deviation

Table 3 shows the secondary endpoints and AEs in each group. The breast lesion and excised specimen marking rates were over 86% in all groups, and there were no significant intergroup differences. However, skin pigmentation was only observed in the control group (n = 9/14, 64.8%, p = 0.000). Histopathological accuracy was measured by a dedicated histopathologist. There were nine cases for which measurement was impossible during histopathologic examination (n = 9/44, 20.4%); there were fibrocystic changes in three cases, inflammation in one case, intraductal papilloma in two cases, and atypical ductal hyperplasia in one case. The mean histopathologic accuracy values in the control group, Test Group 1, and Test Group 2 were 0.3 (range, 0.1–0.9), 0.4 (range, 0.2–0.8), and 0.4 (range, 0.2–0.9), respectively (p = 0.331 between the control group and Test Group 1, p = 0.914 between the control group and Test Group 2, and p = 0.389 between the test groups). No AEs were observed.

**Table 3:** Variables for Secondary End Points and Adverse Events in each Group

	Control group (*n* = 14)	Test Group 1 (*n* = 15)	Test Group 2 (*n* = 15)	*p-value*
Marking on breast lesion				
Yes	13 (92.9%)	13 (86.7%)	14 (93.3%)	*0.954*
No	1 (7.1%)	2 (13.3%)	1 (6.7%)	
Marking on excised specimen				
Yes	13 (92.9%)	14 (93.3%)	15 (100.0%)	*0.357*
No	1 (7.1%)	1 (6.7%)	0 (0.0%)	
Skin pigmentation				
Yes	9 (64.3%)	0 (0.0%)	0 (0.0%)	*0.000*
No	4 (28.6%)	15 (100.0%)	15 (100.0%)	U
Unknown	1 (7.1%)	0 (0.0%)	0 (0.0%)	
Histopathological accuracy				
Mean (±sd)	0.3 (±0.1)	0.4 (±0.1)	0.4 (±0.1)	*0.331(group1-2)* *0.389(group2-3)* *0.914(group1-3)*
Adverse events (AEs)				
Yes	0(0.0%)	0(0.0%)	0(0.0%)	*1.000*
No	14(100.0%)	15(100.0%)	15(100.0%)	

*sd* standard deviation

## Discussion

In this prospective study, we evaluated the efficacy and safety of ICG-hyaluronic acid as a novel agent for localizing non-palpable breast lesions. There were no AEs in any of the groups, and there were no significant intergroup differences in histopathological accuracy, breast lesion marking rates, or excised specimen marking rates. However, there were significant intergroup differences in resection accuracy and skin pigmentation rates.

Excised non-palpable lesions identified by localization could be larger than the excised palpable lesions. The accuracy of resection (the greatest diameter of the excised specimen divided by the greatest diameter of the preoperative lesion as detected by US) reflects the ratio of over-excision. Accuracy values closer to 1 reflect a concordance in size between the ultrasonographically defined lesion and the excised specimen. The mean accuracy value was 3.7 in control group, which was higher than the accuracy value in Test Group 1 and Test Group 2 (p = 0.037 and 0.026, respectively). These differences might have been because the injection dose of activated charcoal (0.3 to 1.0 mL) was much higher than that of the ICG-hyaluronic acid mixture (0.1 mL or 0.2 mL). If activated charcoal had been administered using the same dosage as the ICG-hyaluronic acid mixture, it would have been a proportional comparison. However, if ICG-hyaluronic acid mixture 0.3 to 1.0 mL had been used, localization would have been impossible because of the spread with blur. For the ICG-hyaluronic acid mixture injection, over 60 injections into tissue phantom models (pickle pieces in pork belly) were attempted to determine the appropriate doses and needle thicknesses. The radiologists were technically familiar with and aware of the agents used for localization. Before the implementation of this study, more than 500 breast lesion localizations with charcoal were performed annually at our center. Technical difficulties were not found in the injection of charcoal and ICG-hyaluronic acid mixture. Since a smaller resection could mean a better cosmetic result, the higher resection accuracy in the test groups relative to the control group could have reflected better cosmetic outcomes in the test groups.

Additionally, there was no difference in accuracy between Test Group 1 using 0.1 mL of ICG-hyaluronic acid mixture and Test Group 2 using 0.2 mL of ICG-hyaluronic acid (p = 0.744). Further, there were no differences in the breast lesion marking rates or excised specimen marking rates between the test groups. For localization, ICG-hyaluronic acid could be used at a dosage of either 0.1 mL or 0.2 mL.

The histopathological accuracy (the greatest diameter of the histopathological lesion divided by the greatest diameter of the excised specimen) is a unique variable in our study. Values closer to 1 reflected resections that more closely aligned with the actual histopathological lesions. There were nine cases for which histopathologic measurements were impossible; hence, the sample size was insufficient to assess efficacy in terms of this variable.

The greatest US-measured preoperative lesion diameters were mostly under 2 cm, but the longest diameter was 8 cm in one of the lesions in the control group that was later diagnosed as malignant. The diameter of the excised specimen depended on the histological result, not on the differences between the three groups, which reflects the tendency toward larger margins associated with the resection of malignant vs. benign lesions. If we analyzed only malignant lesions, we would have obtained more accurate results, but this was impossible with so few patients. Our patient group was heterogeneous because only benign lesions were included in the study, but hidden malignancies were discovered after surgery. In the future, we are planning a phase-3 clinical trial to evaluate positive margin rates among cancer patients.

In previous studies, tattooing has been used for various medical purposes [6]. For example, US-guided charcoal tattooing for thyroid cancer has been reported to be safe, easy, and well-tolerated for localizing non-palpable lesions in necks that have previously been operated on, with a high rate of success [7]. For colorectal surgery, preoperative endoscopic tattooing has been demonstrated to be a safe and highly effective method for localization [8]. The use of charcoal for breast marking has been demonstrated to be safe but associated with pigmentation in many studies [9–11]. To our knowledge, no published studies have investigated pigmentation rates in terms of dosage. In our study, skin pigmentation occurred in 64.3% of patients in the control group but in none of the patients in the test groups. Tattooing with charcoal could be removed when the excision includes the skin overlying the lesion [12], but it is impossible in excisions of small lesions. To avoid pigmentation, localization with ICG-hyaluronic acid could be considered. In previous studies, ICG, which was the active component of the ICG-hyaluronic acid mixture, has already been used for medical purposes. For example, the ICG retention rate at 15 min (ICGR15) is a useful marker of liver function in deciding on the extent of hepatectomy [13]. Additionally, Mok et al. reported that ICG-encapsulated hyaluronic acid nanogels could be used for highly selective detection of specific cancers and lymph nodes [14].

Meanwhile, we have presented a new use for ICG-hyaluronic acid. ICG-hyaluronic acid had advantages over activated charcoal in that there was no skin pigmentation and less pain because of the smaller needle (21-gauge versus 18-gauge) used for ICG-hyaluronic acid injection. However, it had a disadvantage in that it could be visualized only via near-infrared fluorescence, so additional equipment was needed.

This study had several limitations. First, the sample size was small, and there was no long-term follow-up. Second, we used only diameter (cm) without volume (cm3 or mL), which better reflects resection margins [15]. Third, errors from individual differences could have occurred, in that four radiologists, five surgeons, and three histopathologists participated in localization, surgery, and histopathologic examination, respectively. Even with these limitations, this study was meaningful because it had a prospective design and because there had previously been no research conducted regarding ICG-hyaluronic acid for breast localization.

## Conclusion

This was a multicenter phase-2 clinical trial to evaluate the efficacy and safety of ICG-hyaluronic acid mixture for localization relative to that of activated charcoal. ICG-hyaluronic acid injection was a new method for localization and useful for obtaining accurate resection as well as cosmetic benefits. Further research is required for the method to be widely used. We have studied a small number of patients with benign breast lesions, but we need to study a larger number of patients with malignant lesions to determine the accuracy and histopathological positive margin rate of this novel method compared with other localization methods. Further research about AEs or survival rates after long-term follow-up should be considered. In the future, we are planning a phase-3 clinical trial to evaluate positive margin rates among cancer patients.

## Data Availability

The datasets used and analyzed during the current study are available from the corresponding author on reasonable request.

## Abbreviations

ICG: Indocyanine green
US: Ultrasound
EKG: Endocardiogram
MMG: Mammography
hCG: Human chorionic gonadotropin
Exam: Examination
AE: Adverse events
pre-OP: Preoperative
ICGR15: The indocyanine green retention rate at 15 min
